# Endothelial nitric oxide synthase gene T^−786^C and 27-bp repeat gene polymorphisms in retinopathy of prematurity

**Published:** 2008-02-05

**Authors:** Krisztina Rusai, Adám Vannay, Beáta Szebeni, Gábor Borgulya, Andrea Fekete, Barna Vásárhelyi, Tivadar Tulassay, Attila J Szabó

**Affiliations:** 1 Research Laboratory of Pediatrics and Nephrology, Hungarian Academy of Sciences; 2 Szentágothai János Knowledge Center, Semmelweis University; 3 Department of Biophysics, KFKI Research Institute for Particle and Nuclear Physics of the Hungarian Academy of Sciences; 4 First Department of Pediatrics, Semmelweis University

## Abstract

**Purpose:**

Retinopathy of prematurity (ROP), which is associated with abnormal retinal vessel development, is the leading cause of visual loss in preterm infants. Endothelial nitric oxide synthase (eNOS) is believed to play a central role in both retinal angiogenesis and vasculogenesis. The aim of this study was to investigate functional genetic polymorphisms of *eNOS* in the pathogenesis of ROP.

**Methods:**

*eNOS* T^−786^C and 27-bp repeat (eNOS, b: wild-type, a: mutant) genotypes were determined using allele-specific polymerase chain reaction in 105 low birth weight (LBW) preterm infants treated for ROP (treated group). A control group was set up and composed of 127 LBW infants with stage 1 or 2 ROP that did not not require treatment (untreated group).

**Results:**

The genotype distribution of *eNOS* 27-bp repeat polymorphism was found to significantly differ (p=0.015) between the two groups, whereas the genotype distribution of *eNOS* T^−786^C did not differ (p=0.984) between the groups. There was no difference in the distribution of either the “a” allele (p=0.153) nor of the C allele (p=0.867) in a groups comparison. Multiple logistic regression analysis revealed that male gender (p=0.046) and *eNOS* aa genotype (p=0.047 versus ab genotype and p=0.022 versus bb genotype) were significantly associated severe ROP that required treatment. The haplotype estimations based on the detected genotype distributions showed that the prevalence of aT and bT haplotypes was significantly increased in the group treated for ROP.

**Conclusions:**

Functional *eNOS* 27-bp repeat polymorphism might be associated with the risk of severe ROP, however we found no association between the *eNOS* T^−786^C and the pathogenesis of ROP.

## Introduction

Retinopathy of prematurity (ROP) is a major cause of blindness in infancy [1]. After preterm birth, the developing retina is exposed to a sudden increase in tissue oxygen tension resulting in the generation of reactive free radicals which may lead to impairment of retinal vascular development and even to loss of already developed retinal capillaries (ROP phase I). This insufficient vascularization results in retinal hypoxia, which, in turn, induces a release of various growth factors, stimulating new and abnormal blood vessel growth (ROP phase II) [2].

Nitric oxide (NO) is a free radical molecule that plays an essential role in numerous physiological actions, including vasoregulation, inhibition of platelet aggregation, and immunological reactions [3]. Endothelial nitric oxide synthase (eNOS), an isoform of NO-producing enzymes that is fairly specific to endothelial cells, has been found to play a prominent role in both angiogenesis and vasculogenesis [4].

NO triggers the gene expression and activation of several angiogenic, cell-migration, and proliferation-inducing factors including fibroblast growth factor 2, vascular endothelial growth factor (VEGF), urokinase-type plasminogen activator, and matrix metalloproteinase [5]. Peroxynitrite, the reaction product of superoxide and NO, is also an important mediator of hyperoxia-induced vaso-obliteration [6].

The expression of eNOS is affected by functional polymorphisms of the *eNOS* gene. Particularly, T^−786^C in the promoter region and 27-bp repeat in intron 4 (*eNOS* b/a) with resultant decreased *eNOS* gene expression have gained more attention [7,8]. These polymorphisms have already been reported to be associated with diabetic retinopathy in type 1 diabetes [9,10] and also with numerous cardiovascular diseases [7,8].

In view of the key role of eNOS in vasculo- and angiogenesis and the association of *eNOS* polymorphisms with diabetic retinopathy, we investigated the association between *eNOS* T^−786^C and *eNOS* 27-bp repeat (b/a) functional polymorphisms and the development of severe ROP in low birth weight infants (LBW).

## Methods

We monitored 232 patients born with LBW (less than or equal to 2000 g) between years 2000 and 2003 for T^−786^C and 27-bp repeat (b/a) *eNOS* gene polymorphisms. They were treated in the neonatal intensive care unit centers of Agost Schöpf-Mérei Institute of Obstetrics, the first Department of Gynecology and Obstetrics, and the second Department of Gynecology and Obstetrics, Semmelweis University, Budapest. All infants enrolled in the study were of Caucasian race. An independent university ethical committee approved our retrospective study (licence No: 16/2003). The research followed the tenets of the Declaration of Helsinki, and informed consent was obtained from the parents to collect blood samples from their children for diagnostic and scientific purposes.

All infants underwent ophthalmologic examination. Maximun ROP stage was assessed and therapy was decided after consultation with two out of the three available neonatal ophthalmologists. The patients were divided into two groups based on requirement for ROP treatment. The first group consisted of 105 infants who had been treated with laser or cryotherapy due to ROP stage 2+ or 5. The mean gestational age was 28±2.5 weeks and mean birth weight was 1150±360 g (treated group).

The second group enrolled 127 preterm LBW infants with ROP stage 1 or 2 who did not require cryotherapy/photocoagulation. The mean gestational age was 30.5±3.5 weeks and birth weight was 1300±400 g (untreated group).

**Figure 1:**
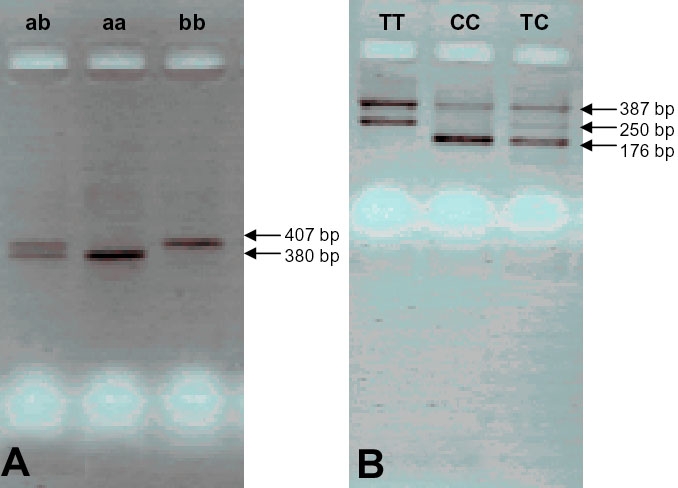
Two represetative pictures of eNOS genotyping. The two pictures show typical results of the allele-specific PCR reactions for *eNOS* 27-bp repeat (**A**) and *eNOS* T^−786^C (**B**) polymorphisms.

DNA for genotyping was extracted from filter papers with an extracting agent (Chelex®; BioRad, Munich, Germany) according to the manufacturers' instructions. *eNOS* T^−786^C SNP was detected using a procedure described by Nasreen et al. [11]. A 27-bp repeat polymorphism was determined using allele-specific PCR with the following conditions: 30 s at 94 °C (denaturing), 60 s at 60 °C (annealing), and 30 s at 72 °C (extension) for 40 cycles. Primer pairs are summarized in Table 1. PCR products were separated on 2.5% agarose gels and visualized under ultraviolet illumination. By the genotyping of *eNOS* T^−786^C, the C and T alleles gave a 176 and 250-bp product, respectively, with a 387-bp common product. PCR amplification of *eNOS* 27-bp repeat polymorphism resulted in a product length of 407-bp (b allele) and of 380-bp (a allele, Figure 1).

**Table 1:** Primer pairs used for allele-specific polymerase chain reaction analysis.

**Gene**	**Primer (5′-3′)**
eNOS T^−786^ C	F: T: CATCAAGCTCTTCCCTGTCT
	R: T0: AGGCCCAGCAAGGATGTAGT

	F: C: GGCAGAGGCAGGGTCAGACG
	R: C0: TTTCTCCAGCCCCTCAGATG

eNOS 27-bp repeat (b/a)	F: TGGGGGAGATCCTTGCCTTTTC
	R: TGAGGGGCTGCACTGGACTGG

Artificially introduced mismatches in the primer sequences are noted in red.

The Harlequin software was used to assess Hardy–Weinberg equilibrium of *eNOS* T^−786^C and *eNOS* 27-bp repeat polymorphisms. The statistical difference, allele frequencies and haplotype distributions among the groups were compared using the chi-square test. Continuous clinical data were compared with Student's *t*-test. Logistic regression analysis was used to assess the association between the need for cryotherapy/photocoagulation and *eNOS* genotypes. The association was adjusted for proven risk factors of ROP [12]: gestational age, days on supplemental oxygen therapy, and their interaction. We perfomed our statistical calculations with the R system [13], using its MASS [14] package.

## Results

Our study enrolled 127 preterm LBW infants with stage 1 or stage 2 ROP who did not require treatment and 105 preterm infants whose ROP required therapy. We assessed their genotypes for the *eNOS* T^−786^C and *eNOS* 27-bp repeat polymorphisms.

The clinical characteristics of the patients are shown in Table 2. Both assessed genotypes were in linkage disequilibrium and in Hardy–Weinberg equilibrium, irrespective of ROP treatment.

**Table 2:** Patient clinical data.

**Clinical characteristics**	**Infants not treated for ROP**	**Infants treated for ROP**	**p values**
Number of patients	127	105	
Males/Females (N)	60/67	67/38	0.0168
Gestational age at birth (weeks)	30.5±3.5	28.4±2.5	0.0001
Birth weight (grams)	1300±400	1150±360	0.003
Days on supplemental oxygen therapy	7 (0–47)	15 (0–92)	0.0001

Gestational age and infant birth weight are shown as mean±SD. The mean and range are provided for days on, supplemental oxygen therapy. In the table, ROP represents retinopathy of prematurity.

Analysis of genotype distributions revealed that the genotype distribution of *eNOS* 27-bp repeat polymorphism was significantly different in the treated group (p=0.015). There was no difference in the genotype distribution of *eNOS* T^−786^C polymorphism compared to the untreated group (p=0.984; Table 3).

**Table 3:** Genotype distribution of *eNOS* 27-bp repeat and *eNOS* T^−786^C polymorphisms two groups of infants with retinopathy of prematurity.

**Gene/region**	**Genotype**	**Infants not treated for ROP (n=127)**	**Infants treated for ROP (n=105)**	**Odds ratio** **(95% CI)**	**p values**
eNOS 27-bp repeat (b/a)	bb	90	60	1.82 (1.14–2.91)	0.015
	ab	36	39		
	aa	1	6		

eNOS T^−786^ C	TT	55	47	0.95 (0.64–1.40)	0.984
	TC	60	79		
	CC	12	9		

The chi^2^ test was used to compare genotype distribution of the different polymorphisms between infants with stage 1 or 2 retinopathy of prematurity (ROP) that was not severe enough to be treated and infants whose severe ROP required treatment. Data are given as the number of patients.

A comparison of the allele frequencies revealed no significant difference in the allele distributions of *eNOS* 27-bp repeat “a” and *eNOS* ^−786^C between the two groups (p=0.153 and p=0.867, respectively; Table 4).

**Table 4:** Allele distribution of *eNOS* 27-bp repeat and *eNOS* T^−786^C polymorphisms in two groups of infants with retinopathy of prematurity.

**Gene/region**	**Allele**	**Infants not treated for ROP (%)**	**Infants treated for ROP (%)**	**Odds ratio (95% CI)**	**p values**
eNOS 27-bp repeat (b/a)	b a	85% 15%	76% 24%	1.79 (0.87–3.66)	0.153
eNOS T^−786^ C	T C	67% 33%	68% 32%	0.95 (0.64–1.40)	0.867

The chi^2^ test was used to compare allele distribution of each polymorphism between the infants whose retinopathy of prematurity (ROP) did not require treatment and infants whose ROP was treated. The frequencies of alleles are given as percentages.

Multiple logistic regression was performed to analyze the relevance of selected parameters (gender, gestational age, time on oxygen therapy and the interaction of gestational age and length of oxygen therapy, and genotypes of *eNOS* 27-bp repeat polymorphism). Results are shown in Table 5. We found that *eNOS* aa genotype and male gender were significant predictors of the onset of ROP requiring treatment among preterm infants (p=0.047 versus ab genotype and p=0.022 versus bb genotype and p=0.046 versus females).

**Table 5:** TResults of multiple regression analysis.

**Variable**	**Odds ratio (95% CI)**	**p values**
Male	1.87 (1.26–4.15)	0.046
Gestational age	0.87 (0.71–1.02)	0.071
Length of oxygen therapy	1.20 (0.99–1.40)	0.082
Gestational age: length of oxygen therapy	0.99 (0.98–1.00)	0.117
eNOS 27-bp repeat aa versus ab	0.10 (−2.13- 2.34)	0.047
eNOS 27-bp repeat aa versus bb	0.08 (−2.12–2.28)	0.022

Models were adjusted by logistic regression analysis for the association with retinopathy of prematurity (ROP) requiring treatment among preterm infants. Association of *eNOS* genotypes was adjusted for gender, gestational age and days of oxygen therapy and for the association of gestational age and days of oxygen therapy.

Based on the genotype distributions, we estimated and compared four haplotypes between the treated and untreated groups. We found that *eNOS* aT and bT haplotypes were significantly increased in the infants treated for ROP compared to the untreated group (p=0.0001 and p=0.0036, respectively; Table 6).

**Table 6:** Results of the haplotype analysis.

**Position**					
**27-bp repeat**	**T^−785^ C**	**Infants not treated for ROP (%)**	**Infants treated for ROP (%)**	**Odds ratio (95% CI)**	**p values**
a	T	7	28	0.19 (0.09–0.45)	0.0001
a	C	17	17	1.01 (0.51–2.00)	0.971
b	T	85	97	0.17 (0.05–0.58)	0.004
b	C	48	47	1.06 (0.63–1.77)	0.94

Data are given as percentages. The comparison was made in infants whose retinopathy of prematurity (ROP) did not require treatment and infants whose ROP was treated.

## Discussion

Several studies have investigated the role of eNOS in connection with ophthalmologic diseases [9,10,15-20]. Different *eNOS* polymorphisms in association with diabetic retinopathy (DR) have been studied extensively. The data are, however, controversial [9,10,18-20]. De Syllos et al. found no association between *eNOS* T^−786^C and Glu298Asp polymorphisms and DR [9]. These results were confirmed by Awata et al. [18]. Yet, a study by Taverna et al. demonstrated that *eNOS* T^−786^C did indeed affect the onset pattern of DR [19]. However, *eNOS* 27-bp repeat polymorphism was not found to be associated with DR in either of the studies [18,20].

This is the first study to investigate the relevance of functional *eNOS* T^−786^C and 27-bp repeat polymorphisms in the pathogenesis of ROP. We found that the genotype distribution of *eNOS* 27-bp repeat was significantly different between the study groups; however there was no difference in the frequency of the “a” allele. Using multiple logistic regression analysis *eNOS* aa genotype was proved to be associated with the onset of ROP requiring treatment. The association was adjusted for ROP risk factors such as gender, gestational age and time on oxygen therapy and for the interaction of gestational age and time on oxygen therapy. We found a significant difference in the birth weight between the two study groups as well, but because there was a strong correlation between the gestational age and birth weight (0.9), we made no adjustment for birth weight.

There was no significant difference in the genotype distribution of *eNOS* T^−786^C polymorphism nor in the frequency of ^−786^C allele between the treated and untreated groups.

Haplotype estimations revealed that prevalence of aT and bT haplotypes was significantly higher in the treated group.

During the first phase of ROP, exposure of the developing retina to relative hyperoxia results in vaso-obliteration in which peroxynitrite, the reaction product of superoxide and NO, has a central role [16].

Brooks et al. showed that oxygen-induced vaso-obliteration in the retina was reduced by administration of an NOS inhibitor or by targeted disruption of the *eNOS* gene in a mouse model, indicating a deleterious role of eNOS-derived NO in the first phase of ROP [15]. Beauchamp et al. found that inhibition of NOS aggravated the retinal vaso-obliteration during exposure to hyperoxia in a rat model [16].

The vaso-obliterative first phase of ROP leads to retinal hypoxia, which, in turn, predisposes to the second phase characterized by abnormal vasoproliferation. Evidence suggests that NO can inhibit angiogenesis under some circumstances, however, present data are controversial.

Mouse implant and chick chorioallantoic membrane models can be used to inhibit, angiogenesis by a NO-donor S-nitroso N-acetyl glutathione [21]. Ando, et al. used *eNOS* knockout mice with ischemic retinopathy and detected, a significant decrease in neovascularization [17]. Interestingly, Campochiaro et al. found that NO in low concentrations triggered the expression of VEGF, which then induced neoangiogenesis [22].

Functional 27-bp repeat polymorphism has a significant role in eNOS production. Tsukada et al. and Hoffmann et al. showed a strong association between eNOS 27-bp repeat polymorphism and plasma NO metabolite levels in healthy subjects [23,24]. Li et al. also reported that NO metabolite levels were lower in those who carry the “a” allele [25]. Indeed, it was demonstrated that nuclear proteins could bind to the 27-bp repeat sequence and therefore decrease gene transcription.

Decreased NO production may aggravate retinal vaso-obliteration during the first phase of ROP [15] and then the neoangiogenesis in the second phase [22]. This could explain the association found in our study between *eNOS* 27-bp aa genotype, leading to decreased eNOS production and severe ROP. However, the serum levels of NO should be determined in order to find out the exact association between *eNOS* 27-bp repeat polymorphism and the risk and severity of ROP.

It is also conceivable that the observed association between 27-bp repeat polymorphism and the severity of ROP is not directly related to NO production capacity. The *eNOS* gene is located near other genes, e.g., T-cell antigen receptor constant beta-chain, which has been shown to be strongly associated with susceptibility to microvascular complications such as retinopathy in type 1 diabetes [26]. Therefore, it is possible that the observed association between *eNOS* 27-bp repeat polymorphism and the severity of ROP is the result of linkage disequilibrium with other gene mutations.

In conclusion, we observed that the genotype distribution of *eNOS* 27-bp repeat polymorphism was significantly different in preterm infants treated for severe proliferating ROP compared to preterm infants infants with stage 1 or 2 ROP that did not require treatment. We also found that *eNOS* 27-bp aa genotype presented an independent risk factor for ROP requiring treatment. On the other hand, no association between *eNOS* T^−786^C and ROP was observed. These findings suggest that *eNOS* 27-bp repeat polymorphism might be associated with the development of proliferative ROP and a indicate the importance of determining the patient's genetic background when planning individual therapy.
